# Functions of the basal lamina and ApoE in cerebral amyloid angiopathy

**DOI:** 10.1186/s12987-025-00734-w

**Published:** 2025-12-12

**Authors:** Alejandra Juan-Palencia, Yao Yao

**Affiliations:** Department of Molecular Pharmacology and Physiology, Morsani College of Medicine, University of South Florida, 12901 Bruce B. Downs Blvd., MDC 8, Tampa, FL 33612 USA

**Keywords:** Cerebral amyloid angiopathy, Amyloid-beta, Basal lamina, Laminin

## Abstract

Cerebral amyloid angiopathy (CAA) is a cerebrovascular disorder marked by the deposition of amyloid-beta (Aβ) peptides within the walls of small- and medium-sized cerebral vessels, including arteries and capillaries but rarely veins. This vascular amyloid burden compromises vessel integrity, causes hemorrhages, and contributes to cognitive decline. Efficient Aβ clearance is critical for preventing its pathological accumulation. Thus, understanding the molecular players within the vascular microenvironment is essential. Laminin, a key glycoprotein of the vascular basal lamina (BL), is fundamental to maintaining structural stability of the vessels and regulating interactions among endothelial cells, pericytes, and the extracellular matrix. However, controversial findings exist on how laminin regulates Aβ aggregation and clearance, with both inhibitory and facilitative effects reported. Genetic variations in laminin subunits, their cell-specific expression pattern, and BL remodeling during CAA further complicate this relationship. This review synthesizes current knowledge on vascular Aβ deposition and elimination in CAA, with a particular focus on the critical roles of the BL/laminin and ApoE in shaping the perivascular microenvironment. First, we introduce Aβ processing relevant to CAA and the mechanisms of Aβ clearance in the CNS. Next, laminin-Aβ interactions and their functions in Aβ clearance are summarized. Thirdly, laminin changes and BL remodeling in CAA are discussed. Finally, we discuss the knowledge gap in the field and fundamental questions that need to be answered in future research. Defining the functions of the BL and ApoE within the pathological context of Aβ-rich vasculature may yield new insights into CAA pathogenesis and reveal therapeutic targets to limit vascular amyloid accumulation. Our goal is to provide a concise review on this matter in order to facilitate new hypotheses in the field.

## Background

Cerebral amyloid angiopathy (CAA) is a cerebrovascular condition distinguished by the accumulation of amyloid-beta (Aβ) in the walls of small- and medium-sized blood vessels (mostly capillaries and arteries but rarely veins), particularly in the cortex and leptomeninges [1]. It is frequently observed in elderly individuals and is also present in Alzheimer’s disease (AD) and Down syndrome, underscoring a pathological link between vascular and neurodegenerative processes [2]. The precise relationship between CAA and AD remains under debate. Some evidence suggests that CAA can occur independently of full AD pathology, as some AD brains lack vascular Aβ deposition despite having widespread plaques and tau pathology [3]. In contrast, other studies argue that CAA may precede and contribute to the development of AD by compromising vascular integrity and Aβ clearance, supporting models such as the “two-hit” hypothesis of AD pathogenesis [4–7]. This ongoing debate reflects the need for deeper mechanistic insight into the state of vascular Aβ over the course of disease progression.

CAA occurs in both sporadic and hereditary forms. Sporadic CAA is driven by vascular Aβ accumulation and is common in aging and some cases of AD. The hereditary form includes rare disorders caused by mutations in the BRI2 gene, such as familial British and familial Danish dementia, both characterized by the deposition of aberrant amyloid peptides [8]. In both types, amyloid deposits are most commonly found in capillaries, arterioles and leptomeningeal arteries. The consequences of Aβ deposition include intracerebral hemorrhage, ischemia, microinfarcts, and progressive cognitive decline [9]. Genetic factors that increase the risk of CAA include mutations in amyloid precursor protein (APP), presenilin-1 (PSEN1), and presenilin-2 (PSEN2); polymorphisms in apolipoprotein E (ApoE); and variants in transforming growth factor beta 1 (TGFβ1), α1-antichymotrypsin (ACT), neprilysin (NEP), low-density lipoprotein receptor-related protein 1 (LRP1), and angiotensin-converting enzyme (ACE) [10]. Histopathological classification of CAA reflects differences in the distribution of vascular Aβ. Type 1 CAA is defined by the presence of amyloid in cortical capillaries, whereas type 2 lacks capillary involvement. These subtypes may be influenced by genetic factors. For example, the ApoE ε4 allele is associated with type 1 CAA and therefore considered as a risk factor for capillary amyloidosis, while the ε2 allele appears more common in type 2 CAA [11].

Although vascular Aβ is the common neuropathological finding, many aspects of CAA pathogenesis remain unclear. The lack of accurate and reproducible animal models continues to limit progress in the field. Most available models depend on the overexpression of mutated APP and present variable degrees of capillary involvement, hemorrhage, or parenchymal plaque deposition. This variability challenges the study of vessel-specific mechanisms and limits the translation of experimental findings to human disease. A better understanding of how Aβ interacts with the basal lamina (BL)---a thin layer of extracellular matrix (ECM) and its major components is necessary to advance the field. Laminins are structural components of the BL that may influence Aβ clearance, deposition, and vascular integrity. This review aims to summarize current knowledge on the relationship between Aβ and BL/ApoE in the context of CAA.

## Main body

### Aβ processing in CAA

Aβ is generated through the proteolytic cleavage of APP, a process mediated by β- and γ-secretases, resulting in a 42- or 40-residue peptide. The human APP gene is a housekeeping gene that encodes a type I transmembrane glycoprotein with multiple functions [12]. Due to alternative splicing, multiple APP isoforms exist with the most abundant ones being APP770, APP751, and APP695 [9, 12]. Aβ must polymerize to form fibrils that deposit in the vessel walls. The rate-limiting step in Aβ polymerization is nucleation, where two Aβ monomers first associate before the polymer extends rapidly toward equilibrium [13]. Several molecules, such as ApoE [11, 14, 15] and laminin [14, 16–21], have been shown to modulate Aβ polymerization/aggregation.

#### Aβ40 and Aβ42

The two major isoforms of Aβ are Aβ40 and Aβ42, which differ in their solubility and aggregation properties. While Aβ40 is more soluble and less prone to form fibrils, Aβ42 is more hydrophobic & fibrillogenic and prone to aggregate [9].

Shifts in the Aβ40:Aβ42 ratio influence whether amyloid mainly accumulates in the brain parenchyma as in AD or in the vasculature as in CAA. Specifically, a higher Aβ40:Aβ42 ratio favors vascular amyloid deposition, as demonstrated in hereditary cerebral hemorrhage with amyloidosis Dutch type (HCHWA-D) patients and APPDutch mice, both of which display almost exclusively vascular amyloid deposition and a significantly elevated Aβ40:Aβ42 ratio [2, 9, 22]. Conversely, an increased proportion of Aβ42 promotes parenchymal amyloid deposition as seen in familial AD cases, where gene mutations shift APP processing toward aggregation-prone Aβ42, resulting in primarily parenchymal amyloid pathology [9, 23, 24]. These findings support the idea that Aβ40, despite being less fibrillogenic, is required for the development of significant vascular amyloid. Nevertheless, it should be noted that Aβ42 may be needed as a nucleating or seeding factor for Aβ aggregation in vascular contexts. Models have shown that Aβ42 is often deposited first, acting as a critical initiator of amyloid formation [25, 26]. Consist with this finding, BRI-Aβ40 transgenic mice, which only express Aβ40, fail to develop amyloid pathology, suggesting that a minimal presence of Aβ42 is necessary to initiate aggregation in vivo [24].

#### Origin of vascular Aβ

In the context of CAA, three potential sources of vascular amyloid have been proposed: neurons, mural cells, and systemic origin [2]. Current literature suggests that the primary source is neurons, which is supported by findings that most APP is produced by neurons and by the success of in vivo models using neuron-specific promoters such as Thy-1 [27].

The mural cell hypothesis suggests that vascular Aβ may originate from these cells through local synthesis. There are two main types of mural cells: smooth muscle cells (SMCs) that line large blood vessels, and pericytes that cover capillaries. Although mural cells have been reported to contribute to the overall Aβ burden [28], there is currently no direct evidence that APP overexpression in SMCs or pericytes causes CAA.

The systemic hypothesis is based on Aβ transport from the plasma into the brain via the RAGE receptor and its clearance through the LRP1 receptor at the blood-brain barrier (BBB) [29]. However, this hypothesis is weak, as in vivo models with systemic APP overexpression fail to develop cerebral amyloidosis, showing neither vascular nor parenchymal Aβ deposits [30]. In sharp contrast, transgenic mice expressing human APP under the neuronal promoter develop CAA [27, 31–35], strongly suggesting that Aβ is primarily produced by neurons and accumulates overtime as a consequence of impaired clearance. Future research should generate mutant mice with APP over-expression exclusively in SMCs, which will help delineate their role in CAA pathogenesis.

#### Models of CAA

Aβ deposition often follows a stereotypical spatial pattern in CAA. Deposition begins in the leptomeningeal vessels, later spreading to cortical, hippocampal, and thalamic vessels, as evidenced in CAA mouse models [27, 31, 32]. At the cellular level, amyloid initially accumulates on the abluminal side of SMCs, facing the brain parenchyma rather than the vessel lumen. As pathology advances, deposits infiltrate between SMCs and ultimately displace or destroy them, rupturing vessels and causing hemorrhages [2].

CAA has been modeled in mice carrying mutations in two major genes: APP and BRI2, which drive vascular deposition of amyloid peptides through distinct mechanisms. Mutations in the APP gene lead to altered production of Aβ. Among the APP mutations studied, the Dutch mutation produces a nearly exclusive vascular Aβ phenotype, as seen in the APPDutch model [31]. This model has been particularly valuable for isolating mechanisms of vascular pathology without the confounding presence of parenchymal plaques. The Swedish mutation results in increased overall Aβ production and widespread vascular amyloidosis as seen in models like APP23 [36], Tg2576 [37] and TgSwDI [32]. Notably, the TgSwDI mice, which combine Swedish, Dutch, and Iowa mutations, exhibit robust cerebrovascular amyloid deposition predominantly in small vessels, modeling type 1 CAA. The London mutation, featured in APP(V717I) [33] and APP(V717I) x PS1 [34], drives both vascular and parenchymal Aβ accumulation, making them appropriate for studying mixed phenotypes. In contrast, models with the Indiana mutation, such as PDAPP [38] and the Arctic mutation, including Arc48 [39] and Tg-ArcSwe [35, 40], predominantly accumulate amyloid in the brain parenchyma with moderate vascular involvement.

On the other hand, mutations in BRI2 (encoding for ITM2B) have been used to model familial Danish dementia, a hereditary form of CAA [8]. These mutations result in the production of amyloidogenic peptides derived from the abnormal processing of the Danish amyloid precursor. The ADanPP and Tg-FDD models carry the Danish mutation and have a prominent vascular amyloid deposition that mimics the human disease phenotype [41, 42]. The BRI-Aβ model, engineered to express Aβ40 or Aβ42 fused to the BRI2 backbone [24], allows for the selective study of specific Aβ isoforms without APP overexpression. These transgenic models represent diverse manifestations of CAA with varying degrees of vascular and parenchymal involvement. Their key features are summarized in Table 1.

**Table 1:** Summary of in vivo CAA models

Key Gene	Model Name	Transgene Construct	Promoter	Age of CAA (m)	CAA Relevance	Reference
APP	APPDutch	APP751 with Dutch mutation (E693Q)	Thy1	~ 22	Pure vascular Aβ, minimal parenchymal plaques	[31]
	APP23	APP751 with double Swedish mutation (K670N/M671L)	Thy1	~ 6–8	Strong parenchymal Aβ, vascular deposition occurs later	[36]
	Tg2576	APP695 with Swedish mutation (K670N/M671L)	PrP	~ 11–13	Strong parenchymal Aβ, some vascular depositions	[37]
	TgSwDI	APP770 with Swedish, Dutch, Iowa mutations (K670N/M671L + E693Q + D694N)	Thy1	~ 3–4	Robust cerebrovascular Aβ in small vessels	[32]
	APP(V717I) ± PS1	APP695 with London mutation (V717I) ± PS1 mutation	Thy1	~ 10	Parenchymal Aβ, vascular depositions after 15 months	[33, 34]
	PDAPP	APP with Indiana mutation (V717F)	PDGF-B	~ 6–9	Primarily parenchymal Aβ	[38]
	Arc48	APP with Swedish, Indiana and Arctic mutation. (K670N/ M671L + V717F + E22G)	PDGF-B	~ 3–4	Primary parenchymal Aβ	[39]
	Tg-ArcSwe	APP695 with Arctic and Swedish mutation (E693G + K670N/M671L)	Thy1	~ 5,6	Strong parenchymal Aβ, moderate vascular plaques	[35, 40]
BRI2	APPDan	Danish BRI2 mutant	Syrian hamster Prion Protein	~ 2	Strong vascular and hippocampal Aβ	[41]
	Tg-FDD	Danish BRI2 mutant	PrP	~ 7	Strong vascular & parenchymal deposition	[42]
	BRI2-Aβ42, BRI2-Aβ40	Fusion protein of BRI2 with Aβ42 or Aβ40	PrP	3 m (BRI2-Aβ42)	Isoform-specific vascular Aβ accumulation	[24]

### Mechanisms of Aβ clearance

**Fig. 1:**
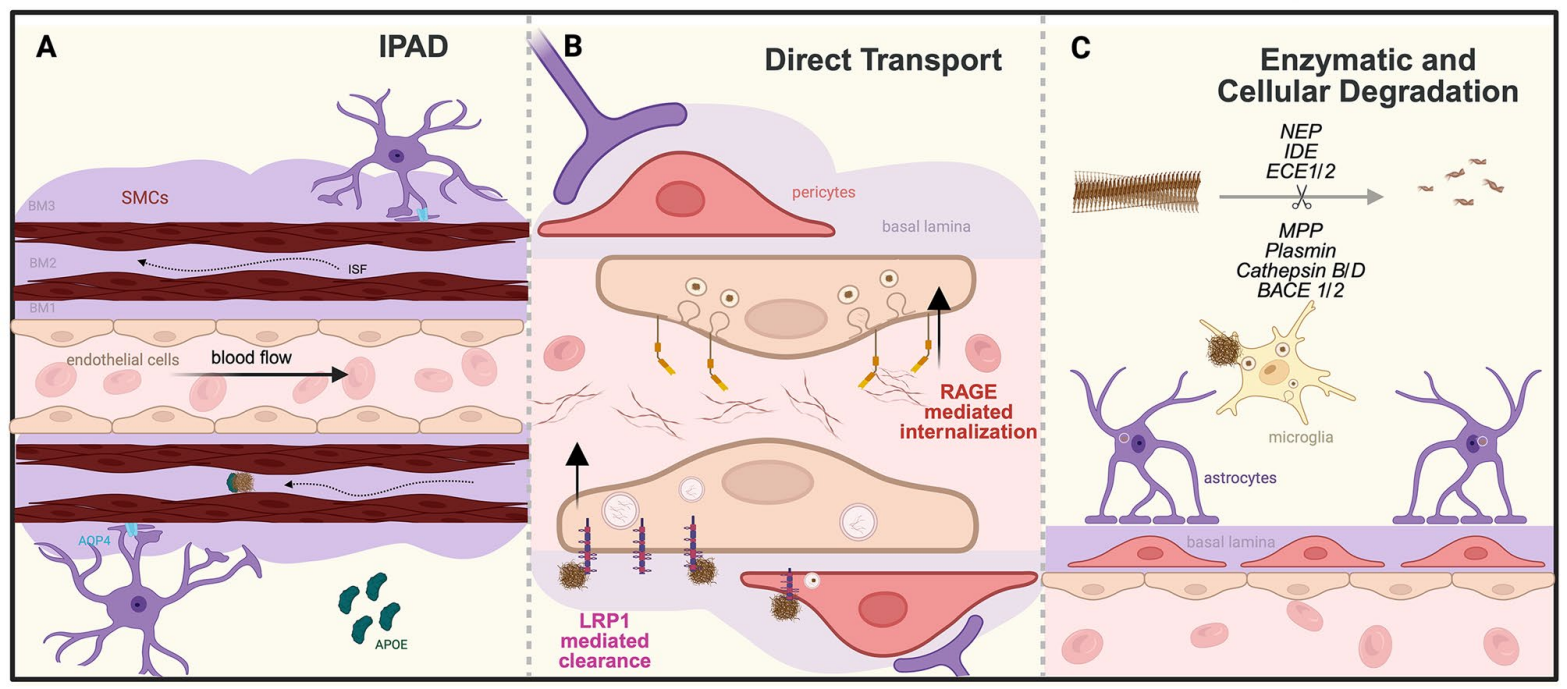
Illustration of three major Aβ clearance pathways. (**A**) IPAD: ISF flows in the opposite direction of blood flow, with Aβ primarily cleared in complexes with ApoE. SMCs regulate vascular dynamics, while astrocytic expression of aquaporin-4 (AQP4) is important for efficient Aβ clearance. (**B**) Direct transport: Efflux of Aβ across the blood-brain barrier is mediated mainly by LRP1 receptor on the abluminal side of endothelial cells, with a minor contribution from pericytes. In contrast, RAGE receptor facilitates Aβ influx from the blood, promoting oxidative stress, BBB breakdown, and mitochondrial dysfunction. (**C**) Enzymatic/Cellular degradation: Major enzymes responsible for Aβ degradation include neprilysin, IDE, ECE1/2, MMP, Plasmin, Cathepsin B/D, and BACE1/2. Major cell types involved in Aβ degradation are microglia and astrocytes. Microglia remove Aβ predominantly through phagocytosis, while astrocytes do so mainly via producing Aβ-degrading enzymes. This figure was made using BioRender

Aβ accumulation could result from excessive production and/or deficient clearance, with the latter getting more attention in the research field [5, 34, 43–46]. It has been reported that Aβ clearance rate is reduced by 30% in AD patients compared to controls, while its production is similar between groups [47]. Aβ is removed from the brain through three main mechanisms: intramural periarterial drainage (IPAD), receptor-mediated transport across the BBB, and enzymatic/cellular degradation [2] (Fig. 1). After IPAD and receptor-mediated transport across the BBB, Aβ binds to molecules in the blood such as immunoglobulins and gets degraded in the liver and kidney [5, 9, 48]. The vascular BL plays a central role in these processes, acting both as a physical duct for clearance and as a modulator of molecular interactions [1, 49–52].

#### IPAD

Aβ is found in the BL of the tunica media of arterioles in CAA and gradually replaces the arterial wall as disease progresses [53]. An important route of Aβ clearance involves its transport along the BL of arteries in a direction opposite to blood flow, facilitated by arterial pulsations, known as IPAD [54]. In the APPDutch mouse model, Aβ is accumulated in vessel walls distant from neurons—its site of production, supporting the idea of a retrograde periarterial transport mechanism [31]. This view aligns with reports describing how Aβ fibrils may move backward toward cerebral arteries, depositing in the arterial walls and culminating in CAA, in parallel with disruption of the clearance system and aquaporin-4 polarization [43]. Additionally, it has been proposed that enhancing perivascular clearance could represent a viable approach to safely reduce Aβ levels in CAA and AD, thereby improving outcomes, such as reduced risk of hemorrhagic stroke and cognitive decline [46].

During this process, Aβ is believed to interact with BL components, such as laminin, to facilitate movement [14]. Accordingly, age-related BL thickening and/or composition changes have been shown to impair this flow, resulting in Aβ entrapment and aggregation [51, 55]. Controversial findings, however, exist on composition changes of the BL during aging. For example, decreased laminin and increased or unaltered collagen IV levels are found in aged human tissue, whereas both increased and reduced laminin and collagen IV levels are reported in aged rodent tissue [20]. This discrepancy may be explained by different tissue origins (humans versus rodents) and/or experimental techniques (IHC or Western blot). In addition, shortcomings in the studies of BL/ECM proteins also contribute to the disparity. For instance, laminin subunit-specific antibodies (e.g. laminin-α4) are frequently used to quantify the expression of specific laminin isoforms (e.g. laminin-α4β1γ1) in Western blot. It is important to know that other laminin isoforms containing the same subunits (e.g. laminin-α4β2γ1) are also quantified, which may complicate data interpretation.

Beyond structural and composition alterations, aging also changes the biochemical modifications of ECM proteins, including non-enzymatic glycation and advanced glycation end-products (AGEs), which may alter ECM stiffness and promote Aβ retention [56]. In addition, transcriptional studies have shown age-associated upregulation of genes involved in ECM turnover, such as matrix metalloproteinases, ADAMTS proteases and their regulators, indicating ECM remodeling [57].

Howe and colleagues proposed five theoretical mechanisms, in which BL remodeling could disrupt Aβ elimination [1]. First, BL remodeling may increase the binding of Aβ to BL components, promoting local retention of Aβ. Second, the structural changes may increase tissue tortuosity, potentially reducing the efficiency of solute movement through the perivascular space. Third, reduced vessel compliance as a consequence of BL stiffening can impair vascular pulsations, which are thought to drive IPAD. Fourth, BL composition changes can impair mural cell function, particularly the contractility of pericytes and SMCs, further reducing vascular dynamics. Finally, remodeling may disrupt aquaporin-4 polarization at astrocyte endfeet, which is essential for waste clearance.

In addition, genetic factors also modulate perivascular clearance of Aβ. It has been shown that ApoE binds Aβ to the vascular BL, enabling its transport along periarterial pathways [14, 58]. This interaction is isoform-dependent: ApoE ε3 binds Aβ more strongly than ε4, facilitating more efficient clearance and reducing aggregation risk [58]. ApoE ε4 forms fewer and less stable ApoE/Aβ complexes compared to ε2 and ε3 [59], a deficit linked to higher levels of Aβ and increased CAA risk consistently observed in ε4 carriers [11, 60]. Consistent with these findings, Bales et al. [61] showed that PDAPP ApoE knockout mice expressing human ApoE ε4 (TRE4) exhibited increased Aβ burden in the brain compared to those expressing ApoE ε2 (TRE2) or ε3 (TRE3) [61], suggesting isoform-dependent effects of ApoE on Aβ accumulation. Additionally, using a similar targeted replacement of human ApoE in mice, Hawkes and colleagues reported that intracerebrally injected human Aβ40 aggregated in the perivascular space of TRE4 mice but not in TRE3 or wild-type mice [62], indicating impaired clearance in ε4 carriers. Analysis of the BL showed that aged TRE4 mice displayed reduced laminin and collagen IV [62], suggesting that ApoE ε4 may promote BL remodeling over time, further compromising Aβ clearance. Collectively, these findings support the view that ApoE isoforms shape perivascular clearance through both biochemical binding properties and structural effects on the BL. While the association between ApoE ε4 and impaired clearance is well supported, the precise molecular mechanisms remain unresolved and need further investigation.

Although less extensive than arterial Aβ deposition, venular Aβ deposition has also been observed. However, there is less evidence implicating veins and venules in Aβ clearance dysfunction, probably because the venous system lacks organized SMCs and has fewer ECM substrates for Aβ anchoring, potentially reducing its clearance capacity [26]. Additionally, the absence of a clear perivascular space and relatively lower blood pressure may also affect Aβ clearance in the venous system [63]. Future research should address this important question.

#### Transport across the BBB

The BBB plays an active role in regulating Aβ levels in the brain. Two major cell types central to this function are endothelial cells and pericytes. Endothelial cells form the barrier and directly mediate Aβ transport [64]. Pericytes support this process by modulating endothelial function and participating in Aβ uptake and clearance themselves [65].

Aβ clearance across the BBB is primarily mediated by LRP1. Located on the abluminal membrane of brain endothelial cells, LRP1 binds Aβ and initiates its movement into the bloodstream [66]. It is estimated that this pathway accounts for approximately 25% of total Aβ clearance in humans [67]. In mouse models, endothelial-specific deletion of LRP1 results in greater Aβ accumulation, confirming its importance [68]. Pericytes also express LRP1 and directly participate in Aβ clearance [65]. In human AD samples, 35% of brain capillary pericytes were found to contain Aβ, and this percentage increased to 60% in a Swedish APP transgenic mouse model [65]. In vitro experiments demonstrated that pericytes clear Aβ42 aggregates through an LRP1-dependent process, which was impaired by knockdown of endogenous mouse ApoE and could be restored by lipidated human ApoE3 but not ApoE4 [65], again highlighting an isoform-specific mechanism.

Several factors influence the efficiency of LRP1-mediated clearance. LRP1 expression has been shown to decrease with age, which may explain the increased Aβ burden in the aging brain [66, 69]. Other regulatory factors include oxidative stress, Srebp2, SLC2A1, and PICALM [66]. Additionally, soluble LRP1 in the plasma can sequester circulating Aβ and support peripheral clearance [66].

On the opposite side of this balance is the receptor for advanced glycation end products (RAGE), which mediates Aβ influx into the brain. RAGE is found on the luminal surface of endothelial cells and facilitates transcytosis of circulating Aβ [29]. This interaction not only increases Aβ entry into the brain but also triggers oxidative stress, mitochondrial dysfunction, and tight junction damage, which further weaken the BBB [29].

Together, endothelial cells and pericytes coordinate Aβ transport across the BBB. Disruption of this balance due to aging, ApoE genotype, or vascular dysfunction may reduce clearance and promote Aβ accumulation, contributing to CAA and/or AD.

#### Enzymatic/cellular degradation of Aβ

A third mechanism of Aβ clearance involves its proteolytic degradation by a diverse set of enzymes termed Aβ-degrading proteases (AβDPs). These enzymes belong to multiple catalytic classes (metallo-, serine-, cysteine-, aspartyl-, and threonine-proteases) and are distributed across various subcellular compartments, including the extracellular space, cytosol, endosomes, lysosomes, and the ER-Golgi network. This spatial heterogeneity means that different pools of Aβ are targeted at distinct stages of Aβ trafficking and accumulation, complicating our understanding of their collective impact on Aβ homeostasis [70].

Among the best-characterized AβDPs are NEP, insulin-degrading enzyme (IDE), and endothelin-converting enzymes 1/2 (ECE1/2). NEP, a zinc metalloprotease primarily active in the extracellular space, is considered the most important endogenous AβDP, with experimental overexpression resulting in up to 90% reduction in brain Aβ levels [71]. Notably, pathogenic Aβ variants, such as those seen in familial AD, often show increased resistance to NEP-mediated cleavage, emphasizing the importance of NEP in disease susceptibility [72]. IDE, found in both the cytosol and extracellular space, is another crucial regulator capable of degrading multiple forms of Aβ, including oligomeric species. ECE1 and ECE2 are localized to acidic compartments and contribute to Aβ degradation in endosomes and the trans-Golgi network [70].

Additional enzymes, such as plasmin (a serine protease), cathepsins B and D (cysteine and aspartyl proteases, respectively), matrix metalloproteases (MMPs), have also been implicated in Aβ degradation. However, their roles remain less clearly defined in vivo. Interestingly, BACE1 and BACE2 (β-secretases), better known for their role in Aβ production, can also contribute to its degradation under certain conditions, blurring the line between production and clearance mechanisms [70]. The irreversible and catalytic nature of Aβ degradation sets it apart from other clearance routes, making AβDPs possible therapeutic targets for reducing cerebral Aβ accumulation in amyloidosis.

In addition to enzymatic degradation, cellular clearance by glial cells is critical for Aβ homeostasis. Microglia, the brain’s resident immune cells, remove Aβ (both soluble and fibrillar forms) primarily through receptor-mediated phagocytosis, although they can also produce AβDPs [73]. Astrocytes contribute to Aβ clearance mainly through the secretion of AβDPs, although they can also phagocytose Aβ [74].

### Laminin-Aβ interactions

The BL is a central hub for Aβ homeostasis. It provides the physical substrate for perivascular drainage, anchors Aβ complexes, and modulates receptor localization at the BBB. BL components, such as laminin, not only maintain structural integrity but also facilitate or hinder Aβ clearance depending on their expression, organization, and molecular interactions [14]. Disruption of these BL components, through aging or disease, may therefore impair multiple clearance mechanisms simultaneously, leading to Aβ accumulation and the onset or progression of CAA. Here, we focus on laminin, a major component of the BL, and discuss its function on Aβ clearance.

#### Laminin

**Fig. 2:**
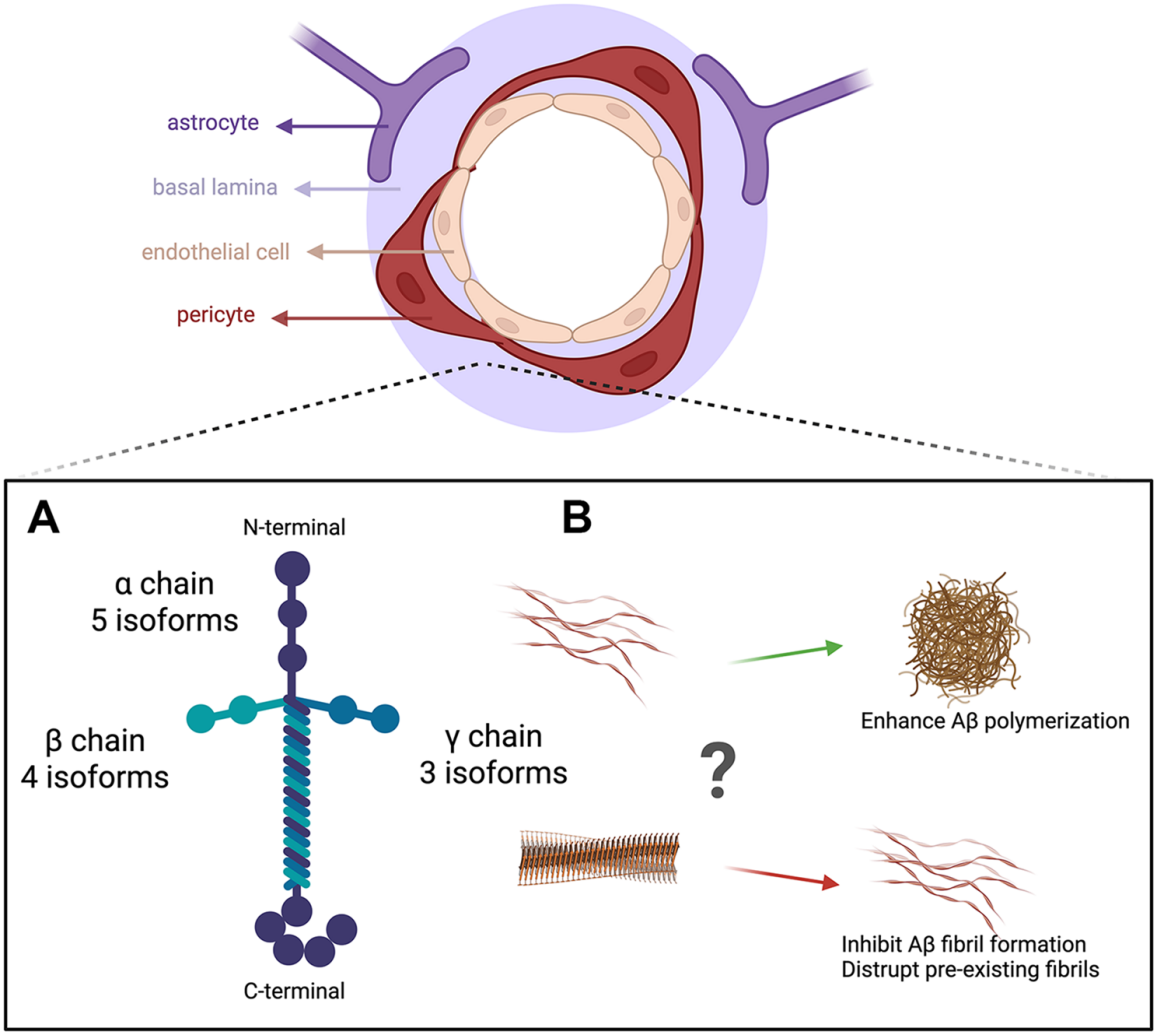
Laminin structure and its function in Aβ polymerization/aggregation. (**A**) Schematic representation of laminin’s structure, a heterotrimeric protein composed of α, β, and γ chains. (**B**) Summary of conflicting findings on laminin’s functions in Aβ polymerization/aggregation. This figure was made using BioRender

Laminin, a T- or cross-shaped glycoprotein, is a key constituent of the BL, which forms a major part of the BBB and the perivascular drainage pathway. Laminin is composed of one α, one β, and one γ subunits, which have 5, 4, and 3 genetic variants in mammals, respectively [75, 76] (Fig. 2A). Although up to 60 laminin isoforms are theoretically possible, only 16 have been experimentally identified, each with potentially distinct functions. In the brain, laminin is mainly produced by endothelial cells, pericytes, SMCs, astrocytes, fibroblasts, and oligodendrocytes [75]. Functional studies using conditional knockout mice demonstrate that laminin is essential for BBB integrity maintenance, with its absence leading to BBB disruption and intracerebral hemorrhages [20, 77]. Its role in neurodegeneration, however, remains controversial. For example, increased, decreased, or unchanged levels of laminin have been reported in AD brains depending on the isoforms and brain regions studied [20, 78]. This discrepancy may be due to different experimental conditions and/or distinct laminin isoforms examined.

#### Laminin-Aβ interactions

Immunohistochemistry has shown that Aβ co-localizes with laminin and other ECM proteins in the brain [1, 50], suggesting that Aβ may interact with laminin. There is evidence supporting a direct binding mechanism between laminin and Aβ via specific motifs. Laminin’s IKVAV sequence has been identified as an important binding site for Aβ [79]. Another Aβ-binding domain has been identified in the α chain near the C-terminal region. Binding affinity assays reveal a dissociation constant (KD) of 2.7nM, indicating a strong interaction between Aβ and laminin [17]. Despite these findings, the downstream effects of this binding remain controversial. On one hand, multiple in vitro studies have suggested that laminin inhibits Aβ polymerization [16–18]. For instance, Drouet et al. (1999) and Castillo et al. (2000) and reported that laminin-111 not only inhibited Aβ fibrillogenesis in a dose-dependent manner, but also disrupted pre-existing fibrils [17, 18]. This inhibitory effect conferred neuroprotection against Aβ-induced toxicity in cultured neurons [18]. Similarly, it was shown that laminin had anti-amyloidogenic properties, capable of both preventing and reversing fibril formation [16]. On the other hand, laminin may also promote Aβ aggregation under certain conditions. It was demonstrated that Aβ polymerized more efficiently in the presence of BL components, including laminin, compared to controls [13]. It remains unclear whether different laminin isoforms have distinct functions in Aβ polymerization/aggregation. The functions of laminin on Aβ polymerization/aggregation are summarized in Fig. 2B.

#### Laminin in Aβ clearance

Laminin may directly interact with Aβ and affect its clearance. Based on the report that laminin-α5 binds to pathological Aβ [19], it is hypothesized that this interaction may help sequester excess Aβ and restore the normal signaling cascades between laminin-α5 and hippocampal neurons. In addition, laminin may also affect Aβ clearance indirectly via mural cells. On one hand, laminin may regulate pericyte biology, which affects BBB integrity and thus Aβ clearance via the BBB route. Yao and colleagues reported that loss of astrocyte-derived laminin-γ1 induces pericyte differentiation from the resting stage to the contractile stage, which impairs BBB integrity [80]. Subsequent in vitro study further demonstrates that laminin’s effect on pericyte biology is mediated by integrin-α2 [80]. Similarly, loss of pericyte-derived laminin-γ1 leads to pericyte defects and BBB breakdown in an age-dependent manner [81]. Although ablation of endothelium- or pericyte-derived laminin-α5 fails to affect pericyte biology or BBB integrity [82, 83], deletion of endothelium- and pericyte-derived laminin-α5 simultaneously results in reduced pericyte coverage and BBB disruption [84]. Consistent with these findings, pericyte defects have been identified as an early event in AD [85–88], which may initiate or promote amyloid vascular accumulation in CAA [89].

**Fig. 3:**
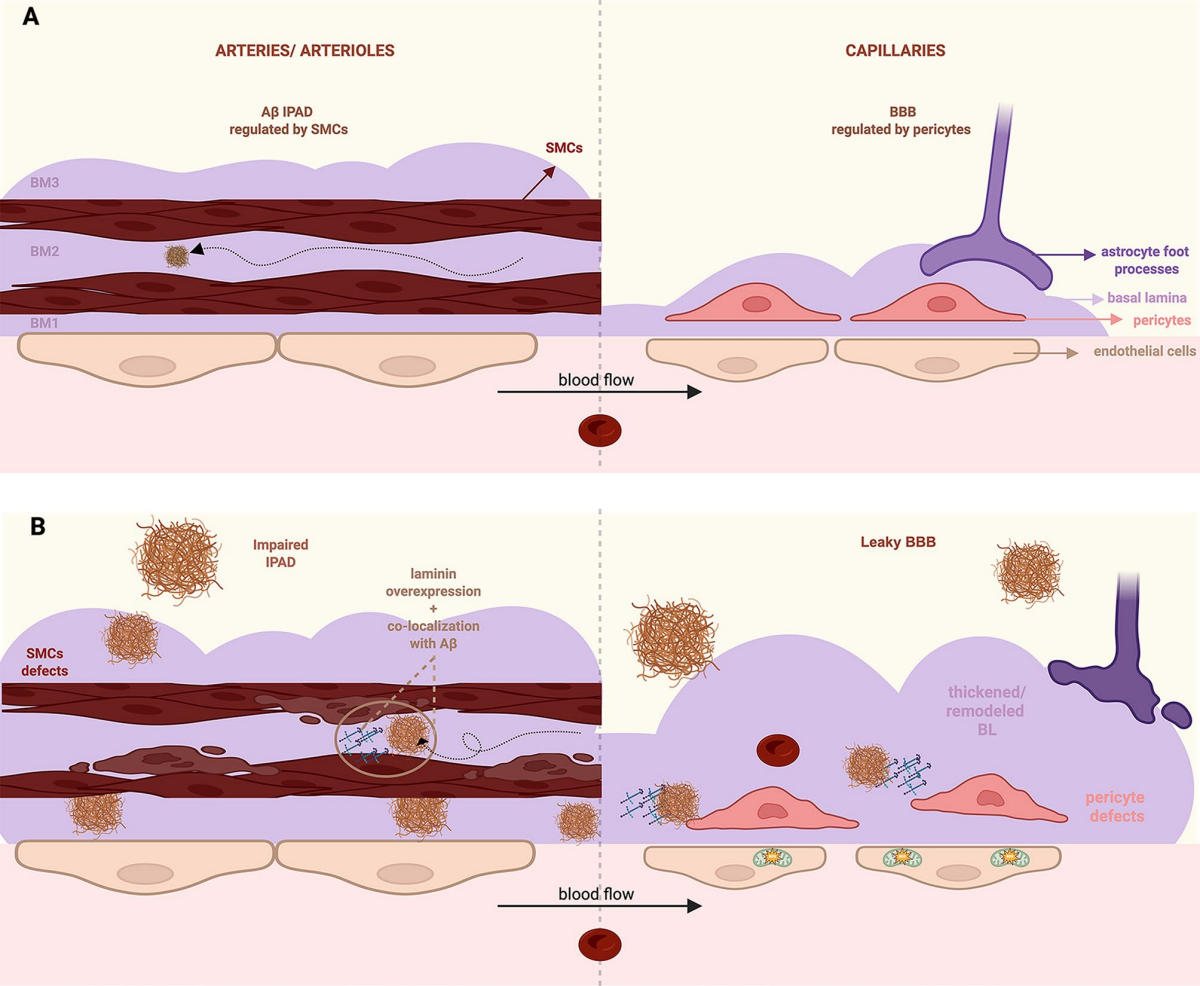
BL/laminin-associated vascular changes in healthy and CAA brains. (**A**) Under physiological conditions, laminin is a key component of the BL, supporting mural cell adhesion and vascular integrity. Aβ is cleared along the periarterial space via the basal lamina, a process partially regulated by SMCs. (**B**) In CAA, laminin is overexpressed and co-localizes with Aβ aggregates within the BL. SMC and pericyte defects contribute to impaired IPAD and BBB disruption, respectively. This figure was made using BioRender

On the other hand, laminin may modulate SMC biology, which affects vasomotion—the rhythmic contractions that drive interstitial fluid (ISF) flow, and thus Aβ clearance via IPAD. It has been reported that ablation of astrocyte-specific laminin-γ1 reduces SMC contractile marker expression and leads to vessel dilation and fragility [90]. Similarly, abrogation of SMC-derived laminin-γ1 results in abnormal SMC morphology and reduced blood pressure [91]. These findings suggest that laminin participates in the regulation of SMC contractility and its dysregulation may contribute to Aβ accumulation [1]. Interestingly, Aβ40 significantly reduces the adhesion of vascular SMCs to laminin in vitro, which may contribute to cell degeneration and vessel wall weakening in CAA [92]. Together, these results highlight a potentially bidirectional relationship: while mural cells synthesize and deposit ECM proteins to the BL, BL changes may in turn impair mural cell functions and Aβ clearance. Laminin/BL changes at the arteries/arterioles and capillaries in CAA are summarized in Fig. 3.

### Laminin changes and BL remodeling in CAA: mediator or consequence?

Laminin and collagen IV show divergent expression patterns in AD and CAA. It has been reported that the levels of both proteins are reduced in AD and elevated in CAA, compared to healthy controls [3]. Immunohistochemistry studies have consistently shown that laminin expression co-localizes with vascular Aβ deposits in CAA brains [50]. It remains unclear whether this reflects a pathological interaction or a protective attempt at structural reinforcement.

A temporal study in the arcAβ mouse model showed increased laminin intensity prior to substantial amyloid deposition [40]. In advanced stages, large vessels with severe CAA exhibited pronounced distortions, such as constrictions and ruptures, and correlated with increased laminin intensity [40]. Since mural cells in large vessels also synthesize laminin and contribute to the elevated laminin intensity, it remains unclear whether increased laminin levels cause amyloid accumulation and vascular degeneration in these mice. Further research is required to demonstrate causality.

Additionally, BL thickening and collagen IV accumulation have also been reported in other small vessel disease models with vascular Aβ deposition, supporting the notion that BL remodeling might not only reflect pathology but also contribute to its initiation [93]. In fact, impaired Aβ clearance, potentially driven by altered BL structure, is increasingly viewed as an early event in CAA and possibly a driver of AD pathology as well [5]. Notably it has been demonstrated in vivo that ApoE isoforms modulate BL composition over time, with ApoE ε4 carriers exhibiting reductions of laminin and collagen IV at later stages [62], suggesting that ApoE ε4 influences laminin remodeling in a manner that may exacerbate vascular dysfunction and impaired clearance in CAA.

## Conclusions and future directions

With advances in genetic and biomedical techniques, substantial progress has been made in our understanding of CAA pathogenesis. However, several fundamental questions remain unanswered and need future research.

First, laminin modulates both the biochemical properties of Aβ and the mechanical properties of vessel walls, and thus is well positioned to regulate Aβ aggregation and clearance. However, the physiological impact of isoform diversity and cell-specific expression of laminin in the CNS remain incompletely understood. Future research should elucidate cell-specific sources of laminin isoforms in the cerebrovasculature and define their functional significance in physiological and pathological conditions.

Next, how laminin regulates Aβ biochemistry in vitro remains largely unknown. While existing studies show that laminin can alter Aβ polymerization, aggregation, and fibril morphology, most experiments rely on tumor cell-derived laminin-111. This isoform is expressed by fibroblasts at low levels in the healthy adult brain [19], raising concerns about the physiological relevance of these findings. Clarifying how brain-relevant laminin isoforms influence Aβ structure, solubility, aggregation, and clearance will help determine whether laminin acts in a protective or pathological capacity at the molecular level.

Third, the mechanisms by which the BL influences Aβ deposition and clearance in vivo remain poorly defined. Whether and how laminin directly interacts with Aβ or alters BL properties to affect clearance efficiency are unknown. Additionally, it remains unclear if laminin acts primarily as a scaffold that traps Aβ40 in vessel walls, thereby accelerating vascular deposition, or if its upregulation is a reactive change aimed at reinforcing damaged BL. Distinguishing these possibilities is crucial, as it will determine whether targeting laminin represents a viable therapeutic strategy or risks destabilizing vascular integrity. Developing in vivo models with targeted manipulation of laminin isoforms or cell-specific expression will be critical to unravel these mechanisms.

Additionally, how other BL components contribute to Aβ deposition and clearance remains largely unexplored. Collagen IV, perlecan, and nidogen may cooperate or compete with laminin to shape the vascular BL structure and function. BL composition changes with age and injury, so mapping its remodeling over CAA progression could reveal early pathogenic events and intervention windows. Interaction of Aβ with these components forms a dynamic scaffold that might play a role in Aβ’s fate. In vivo models with precise genetic tools will be crucial to clarify how these interactions influence vascular amyloid load.

Last, it remains unknown how other genetic risk factors modulate laminin-Aβ interaction, although ApoE isoforms are involved in this process. The effects of other genetic risk factors on laminin-Aβ interaction should be determined in future research.

Addressing these challenges will refine our understanding of CAA pathogenesis and may uncover novel therapeutic targets to slow or prevent vascular amyloid deposition.

## Data Availability

No datasets were generated or analysed during the current study.

## Abbreviations

Aβ: β-amyloid
AβDPs: Aβ-degrading proteases
ACE: Angiotensin-converting enzyme
ACT: α1-antichymotrypsin
AD: Alzheimer’s disease
ApoE: Apolipoprotein E
APP: Amyloid precursor protein
BBB: Blood-brain barrier
BL: Basal lamina
CAA: Cerebral amyloid angiopathy
ECE1/2: Endothelin-converting enzymes 1/2
ECM: Extracellular matrix
HCHWA-D: Hereditary cerebral hemorrhage with amyloidosis Dutch type
IDE: Insulin-degrading enzyme
ISF: Interstitial fluid
IPAD: Intramural Periarterial Drainage
LRP1: Low-density lipoprotein receptor-related protein 1
MMPs: Matrix metalloproteases
PSEN1/2: Presenilin-1/2
RAGE: Receptor for advanced glycation end products
SMCs: Smooth muscle cells
TGFβ1: Transforming growth factor beta 1
